# Protection by beta-Hydroxybutyric acid against insulin glycation, lipid peroxidation and microglial cell apoptosis

**DOI:** 10.1186/s40199-015-0126-5

**Published:** 2015-08-27

**Authors:** Manijheh Sabokdast, Mehran Habibi-Rezaei, Ali Akbar Moosavi-Movahedi, Maryam Ferdousi, Effat Azimzadeh-Irani, Najmeh Poursasan

**Affiliations:** School of Biology, College of Science, University of Tehran, Tehran, Iran; Nano-Biomedicine Center of Excellence, Nanoscience and Nanotechnology Research Center, University of Tehran, Tehran, Iran; Institute of Biochemistry and Biophysics, University of Tehran, Tehran, Iran; Center of Excellence in Biothermodynamics, University of Tehran, Tehran, Iran; Present address: Department of agronomy, and plant breeding, College of Agriculture & Natural Resources, University of Tehran, Karaj, Iran

## Abstract

**Background:**

Diabetes mellitus is characterized jointly by hyperglycemia and hyperinsulinemia that make insulin more prone to be glycated and evolve insulin advanced glycation end products (Insulin- AGE). Here, we report the effect of beta-hydroxy butyrate (BHB) (the predominant ketone body) on the formation of insulin-AGE, insulin glycation derived liposomal lipid peroxidation and insulin-AGE toxicity in microglial cells.

**Methods:**

The inhibitory effect of BHB was monitored as a result of insulin incubation in the presence of glucose or fructose using AGE-dependent fluorescence, Tyr fluorescence as well as anilinonaphthalenesulfonate (ANS) andthioflavin T (ThT) binding, and circular dichroism (CD) investigations. To study lipid peroxidation induced by insulin glycation, thiobarbituric acid (TBA) assay and thiobarbituric acid reactive substance (TBARS) monitoring were used. The effect of insulin–AGE on microglial viability was investigated by 3-(4, 5 dimethylthiazol-2-yl)—2, 5-diphenyltetrazoliumbromide (MTT) cell assay and Annexin V/propidium iodide (PI) staining.

**Results:**

Here we are reporting the inhibitory effect of BHB on insulin glycation and generation of insulin-AGE as a possible explanation for insulin resistance. Moreover, the protective effect of BHB on consequential glycation derived liposomal lipid peroxidation as a causative event in microglial apoptosis is reported.

**Conclusion:**

The reduced insulin fibril formation, structural inertia to glycation involved conformational changes, anti-lipid peroxidation effect, and increasing microglia viability indicated the protective effect of BHB that disclose insight on the possible preventive effect of BHB on Alzheimer’s disease.

## Introduction

Type 1 Diabetes is generally characterized by raised level of blood sugar (hyperglycemia) due to imperfection in insulin secretion, type 2 diabetes is characterized by insulin resistance that results in both of hyperglycemia and hyperinsulinemia and finally type 3 diabetes is characterized with neurodegeneration linked with insulin resistance. Although, increasing evidences in the literature pointing toward strong correlation between insulin resistance and Alzheimer’s disease (AD) [1, 2] but, that correlation has not been yet formally recognized.

Under hyperglycemic condition almost all proteins are prone to be glycated in a nonenzymatic fashion. It is established that protein glycation, oxidative stress, and lipid peroxidation are key processes in diabetes and related complications [3, 4]. Due to the glucose auto-oxidation and protein glycation, hyperglycemia result in increased production of reactive oxygen species (ROS) that originates oxidative stress as an imbalance between radical- generating and radical-scavenging systems [5].

Protein glycation is whereby labile Schiff base is formed by nonenzymatic reaction between annomeric carbonyl group of an open ring carbohydrate and amino group(s) of the protein molecule followed by molecular rearrangement to form stable Amadori products that provoke the formation of advanced glycation end products (AGE) after additional dehydration reaction and further molecular rearrangements [6]. Following the protein glycation, a series of events occur covering α-helix to β-sheet transformation, cross β structure formation, and generation of soluble amyloid prefibrils [7]. Membrane lipids are mainly prone to oxidation by ROS owing to their polyunsaturated fatty acid content [8]. That is why prefibriles and ROS initiate membrane involved events or damages, that consequently induce apoptotic response and cell death [9]. Hence, antioxidants suppose to effectively protect against glycation derived free radicals and considered as a therapeutic potential for the inhibition of ROS involved processes [10].

Ketone bodies (KB) comprise 3-beta-hydroxybutyrate (BHB), acetoacetate (AcAc), and acetone while the later is the least abundant one. They are always present in the blood and their levels increase during fasting and prolonged exercise [11]. They are also found in the blood of neonates and pregnant women. However, type 1 diabetes is the most common pathological cause of raised blood ketones due to increased lipid catabolism under hypoinsulinemia. In such a condition, the BHB: AcAc ratio arises from normal, 1:1 to as high as 10:1. It has been a decade that BHB is reported to be useful against cell apoptosis [12], adipocyte lipolysis inhibition [13], and considered as a treatment for various diseases including epilepsy, Huntington’s, Parkinson’s, and Alzheimer’s [14, 15]. Concurrency of hyperglycemia and hyperinsulinemia in type 2 diabetes, make insulin prone to be glycated. Here we are reporting the inhibitory effect of BHB on insulin glycation and generation of insulin advance glycation end product (insulin-AGE) as a possible explanation for insulin resistance. Moreover, the protective effect of BHB on consequential glycation derived liposomal lipid peroxidation as causative events in microglial apoptosis are reported.

## Materials and methods

### Material

Newborn rats (Wistar strain) were obtained from the University of Tehran animal facilities. The Annexin-V-FITC apoptosis assay kit was from Molecular Probes Inc., UK. Human recombinant insulin was gifted by Exir pharmaceutical company (Iran). Thioflavin T was from Merck Company and other chemicals used in this study were obtained from Sigma Aldrich (USA). All solutions were prepared using double—distilled water.

### Sample preparation

Insulin at a final concentration of 0.5 mg.ml^−1^ was dissolved in phosphate buffer (50 mM, pH 7. 4) and incubated with 16.5 mM D-glucose or D-fructose in either the presence or absence of 14.4 mM β-hydroxy butyrate (BHB) which is close concentration in individuals with post prolonged fasting (mM) or ketoacidosis, based on literature [16–19]. All solution was filtered using 0.2 μm membrane filter (Milipore, USA) under sterile condition. All samples were incubated under physiological conditions (at dark and 37 °C) for 0 to 96 h, and then stored at −20 °C until using for further analysis.

### Fluorescence measurement

The Cary Eclipse fluorescence spectrophotometer (Varian, Australia) was used for monitoring the AGE dependent fluorescence, the changes in the environment of the Tyr residue in insulin and for probing the available hydrophobic portion of protein (ANS) and ELISA reader fluorescence H4 (Synergy H4, Bio Tek, USA) was used for ThT binding analysis. Protein intrinsic (Tyr) fluorescence was analyzed at 307 nm after excitation at 276 nm. AGE dependent fluorescence intensity measurement of glycated insulin in the presence or absence of BHB was carried out at 384 nm excitation wavelength and emission spectra were recorded in the wavelength range of 384–500 nm. The protein concentration was 0.5 mg.ml^−1^.

For ANS fluorescence measurement, 200 μL from the incubated mixtures (0.2 mg.ml^−1^ insulin) in the presence or absence of BHB (14.4 mM) at 37 °C were added to fresh solution of ANS (3 μM in 50 mM phosphate buffer pH 7.4) and incubated for 30 min in the dark, then the emission intensity was measured after excitation at 463 nm at room temperature to study the kinetics of change in solvent-exposed hydrophobic pockets due to the generation of partially folded intermediates during insulin glycation, aggregation, and AGE formation. For ThT assay 20 μL aliquots of 0.5 mg.ml^−1^ samples were added to 180 μL solution containing 25 μM ThT (in 50 mM phosphate buffer pH 7.4), then fluorescence emission was determined at 490 nm using a H4 spectrometer. Fluorescence emission intensities were plotted against time after excitation at 440 nm. The emission values of the buffer and fresh insulin were used as background correction and control, respectively. The averages of triplicate measurements were used for each sample.

### Circular dichroism (CD) spectroscopy

Far-UV CD was used for analyzing secondary structure during fibrillation. The CD measurements were obtained using a CD spectropolarimeter (J-810, Jasco, Japan) with 1-mm path length of a quartz cuvette at 25 °C and data were scanned from 190 nm to 260 nm at 1 nm intervals. The final protein concentration was 0.2 mg.ml^−1^in 50 mM phosphate buffer pH 7.4. The bandwidth was set at 1 nm. The spectrum of phosphate buffer was subtracted from sample spectra for data analysis. All CD spectra converted to mean residue ellipticity using the following relationship:

$$ \left[\uptheta \right] = \left(\uptheta /10\right)\ \left(\mathrm{M}\mathrm{R}\mathrm{M}/\mathrm{L}\mathrm{C}\right) $$

Where [θ] is ellipticity (deg.cm^2^.dmol^−1^) at wavelength λ, θ is the observed ellipticity in millidegree, MRW is the mean residue weight, L is the path length (in cm), and C is the protein concentration (mg.ml^−1^). The percentage of secondary structure was obtained using CDNN software.

### Preparation of liposome and lipid peroxidation assay

Liposomes were made using a modification of the method of Bangham [20]. Briefly, a solution of soybean phosphatidylcholine/cholesterol in the weight ratio of 4:1 in chloroform was dried under reduced pressure using a rotary evaporator at <50 °C to provide a thin homogenous film, which was placed in a desiccator for next 24 h. The film was then dispersed in phosphate buffer and stirred for 15 min. The mixture was sonicated to achieve a homogeneous suspension of liposomes. Lipid peroxidation was used as an indicator of tissue injury induced by reactive oxygen species. It was measured using the thiobarbituric acid assay (TBA) based on thiobarbituric acid reactive substance (TBARS) monitoring. The amount of tissue TBARS was measured by the method described by Buege and Aust [21]. In brief, 250 μl of sample at incubation time periods (10, 30, 50, 70, and 90 h) was added to an aliquot of liposomes and then incubated at 37 °C for 24 h. Then 50 μL of TCA (50 %) and 100 μL of TBA (0.35 g) were added to the reaction mixture. It absolutely was then incubated for a quarter-hour at boiling water bath and TBARS was identified at 532 nm.

### Cell culture and cell viability assay

The microglia from neocortex of newborn rats (Wistar strain) were cultured from mixed glia cultures according to the procedure by Giulian and Baker with some modifications [22]. Briefly, the cerebellum was detached, meninges were dissected, and brain cortex tissue was minced in a nutrient medium. Then cells were dissociated by triturating with sterile pipettes to obtain a cell suspension. The cell suspension from each brain was separated into two 75 ml tissue culture flasks (Falcon) in DMEM and 20 % FCS at 37 °C with 5 % CO_2_ for 24 h. After 24 h, the medium was half changed to reach 10 % FCS for the rest of the day. The cells were fed every 4 days with a fifty percent spent medium. After 2 weeks, cultures contained glial cells, including rounded microglial cells mostly localized on the top of the monolayer. The loosely adherent microglial cells were recovered by gentle shaking by hand for 2 min. The cell suspension was then cultured on 96 multiwell plates at a density of 3 × 10^4^ cell/cm2, in 10 % FCS supplemented DMEM medium (total volume 200 μl) for 24 h, to enter the ramified phase. To treat the cells, the culture medium was replaced with the insulin (6.25, 12.5, 25, 50, 75,125, 200, and 375 μg/ml, the final concentration of the glycated insulin was determined according to the final volume of 200 μl) which was glycated in the presence or absence of BHB for a series of incubation time (0, 10, 30, 50, 70, and 90 h). Treated cells were kept in this medium for 24 h, after which the effect of AGE on the viability of the cells was evaluated via the MTT assay [23]. This assay measures the mitochondrial function and is most frequently used to detect loss of cell viability [24]. Nevertheless, this assay can underestimate the cell death because it works best to detect the later stages of apoptosis when the metabolic activity of the cells is strictly reduced [25]. The treated cultured cells were incubated with 10 % of MTT per well for four hours (from the stock solution of 5 mg.ml^−1^ of MTT in PBS, which was filtered through 0.2 μm syringe filter and kept all the time in dark condition), after which the whole media were replaced with 100 μl DMS solution to dissolve the MTT formazan crystals. The optical density (OD) at 580 nm was determined using an EIA Multiscan MS micro-plate reader.

Cell survival was calculated as a percentage by dividing the absorbance values of the experimental group (treated cells) by the absorbance values of the control group (untreated cells). Each assay was repeated six times, to ensure the reproducibility of the results. Moreover, apoptosis analysis was performed using Annexin V-FITC and propidium iodide (PI) dual staining according to the manufacturer’s instructions. Briefly after a period of treatment, cells were harvested and washed in cold phosphate-buffered saline (PBS) followed by centrifugation at 900 × g for 10 min. The pellet re-suspend in 200 μL annexin binding buffer to prepare a cell density of 1 × 10^6^ cell.ml^−1^. Then 5 μl of Alexa Fluor 488 annexin V and 5 μl of the 50 μg.ml^−1^ propidium iodide (PI) solutions was added. After 15 min incubation at room temperature, 300 μl of 1× annexin binding buffer was added and samples were kept on ice. Then the cells were analyzed by flowcytometry, measuring the fluorescence emission at 530 nm (e.g. FL1) and 575 nm (e.g. FL2). After staining apoptotic, necrotic and live cells show green, red and no fluorescence, respectively [26]. All procedures were performed in accordance with institutional guidelines for animal care and use, which adhered to the international principles of Laboratory Animal Care (NIH publication #85-23, revised in 1985).

### Statistical analysis

Data were expressed as mean ± SD. static analysis between treatments was made using one-way ANOVA (analysis of variance) followed by Duncan’s new multiple range tests for multiple comparisons. P-value <0.01 was considered statistically significant.

## Results

**Fig. 1:**
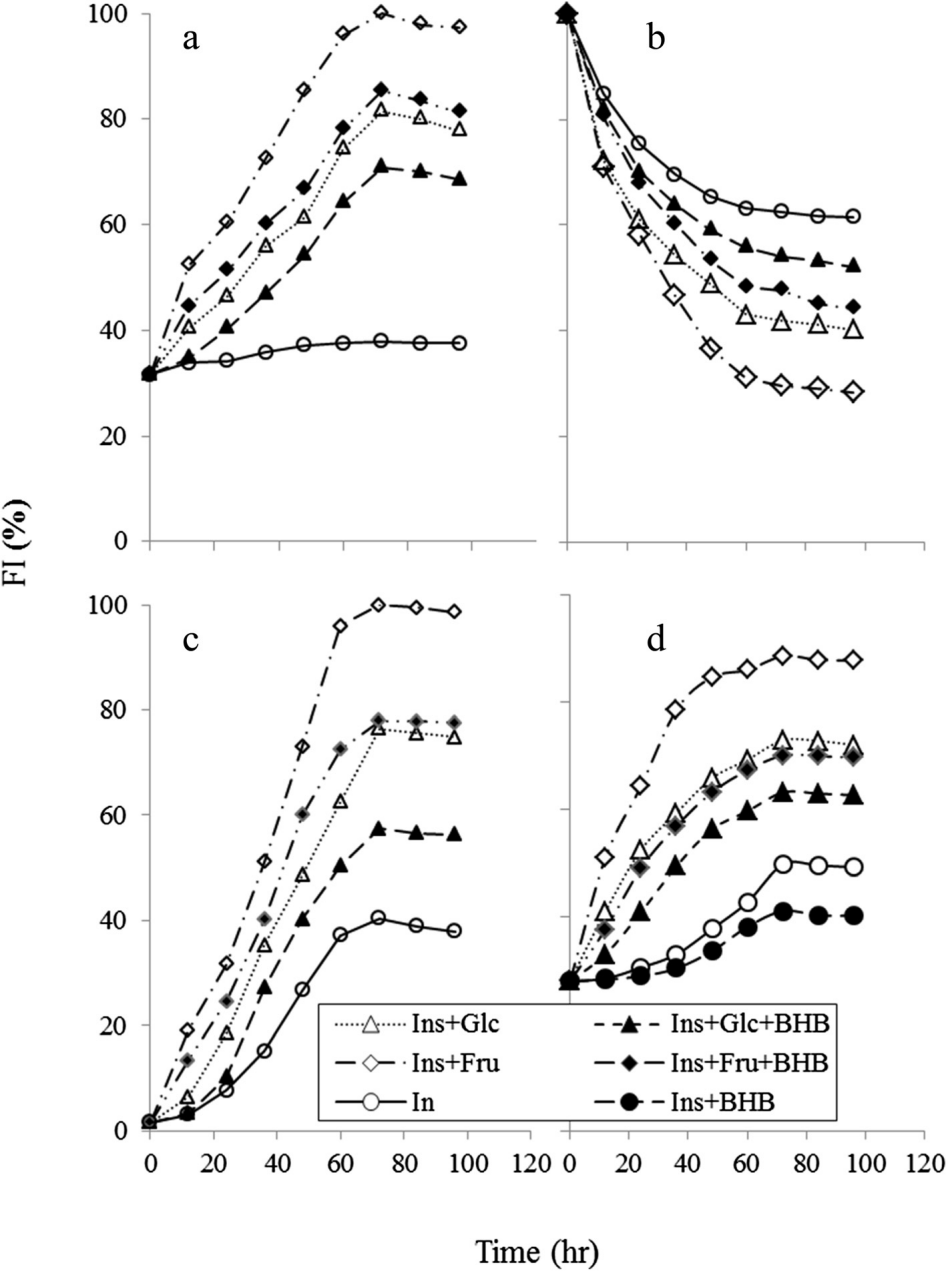
The kinetics of changes in the fluorescence of glycated insulin in the presence or absence of BHB. **a** AGE-related auto-fluorescence of insulin and modified insulin (Ins + Glc, Ins + Glc + BHB, Ins + Fru, Ins + Fru + BHB) was monitored in emission wavelength range of 384–500 nm. After excitation at 370 nm. Insulin, glucose, fructose and BHB were alone used as a control. **b** Changes in the intrinsic fluorescence (λex 276 nm; λem 307 nm) of insulin incubated with Glc or Fru in the presence or absence of BHB (**c**) Extrinsic ANS fluorescence, was used to assess the change in surface hydrophobicity of insulin. Aliquots of the incubated insulin were added to 3 μM ANS and the spectra recorded after 30 min. The excitation was performed at 350 nm and fluorescence spectra were obtained from 405 nm to 550 nm. **d** The β-sheet content of insulin was determined with Thiflavin T fluorescence. Excitation and emission wavelength was 450 nm and 490 nm respectively

Intrinsic and extrinsic fluorescence analysis Conformational change, the formation of glycation products, and increasing surface hydrophobicity of insulin alone as a control, insulin in the presence of Glc or Fru and in the presence or absence of BHB were examined using intrinsic tyrosine (excitation at 280 nm), AGE-dependent (excitation at 384 nm), and ANS-binding fluorescence (excitation at 460 nm), respectively (Fig. 1). Fig. 1a illustrates increase in the intensity of AGE-dependent fluorescence (λ_ex_ 370 nm;λem425 nm) to detect the formation of AGE [27] upon glycation by Glc or Fru also the inhibitory effect of BHB has been presented. The kinetics of Tyr fluorescence of insulin under glycation in the presence of Glc or Fru and presence or absence of BHB has been depicted in Fig. 1b. Fluorescence at 280 nm region is commonly used to study conformational alterations of a protein in solution [28]. The intrinsic fluorescence of insulin decreased markedly during incubation with glucose or fructose that was diminished by BHB which indicates an inhibitory effect of BHB on glycation-induced insulin conformation change (Fig. 1b). ANS fluorescence was employed to study the kinetics of change in solvent exposed hydrophobic pockets of insulin in the presence of insulin and Glc or Fru and presence or absence of BHB to characterize partially folded intermediates during insulin glycation, aggregation, and AGE formation (Fig. 1c). The fluorescence intensity enhancement of ANS in glycated insulin with glucose or fructose indicates the increase in solvent-exposed hydrophobic regions, originating from partially folded intermediates. BHB was approved to decrease glycation-involved ANS fluorescence.

Thioflavin T fluorescence test resulted less β-sheet content in the samples glycated by Glc or Fru in the presence of BHB than the samples in the absence of BHB (Fig. 1d).

### Circular dichroism (CD) analysis

**Fig. 2:**
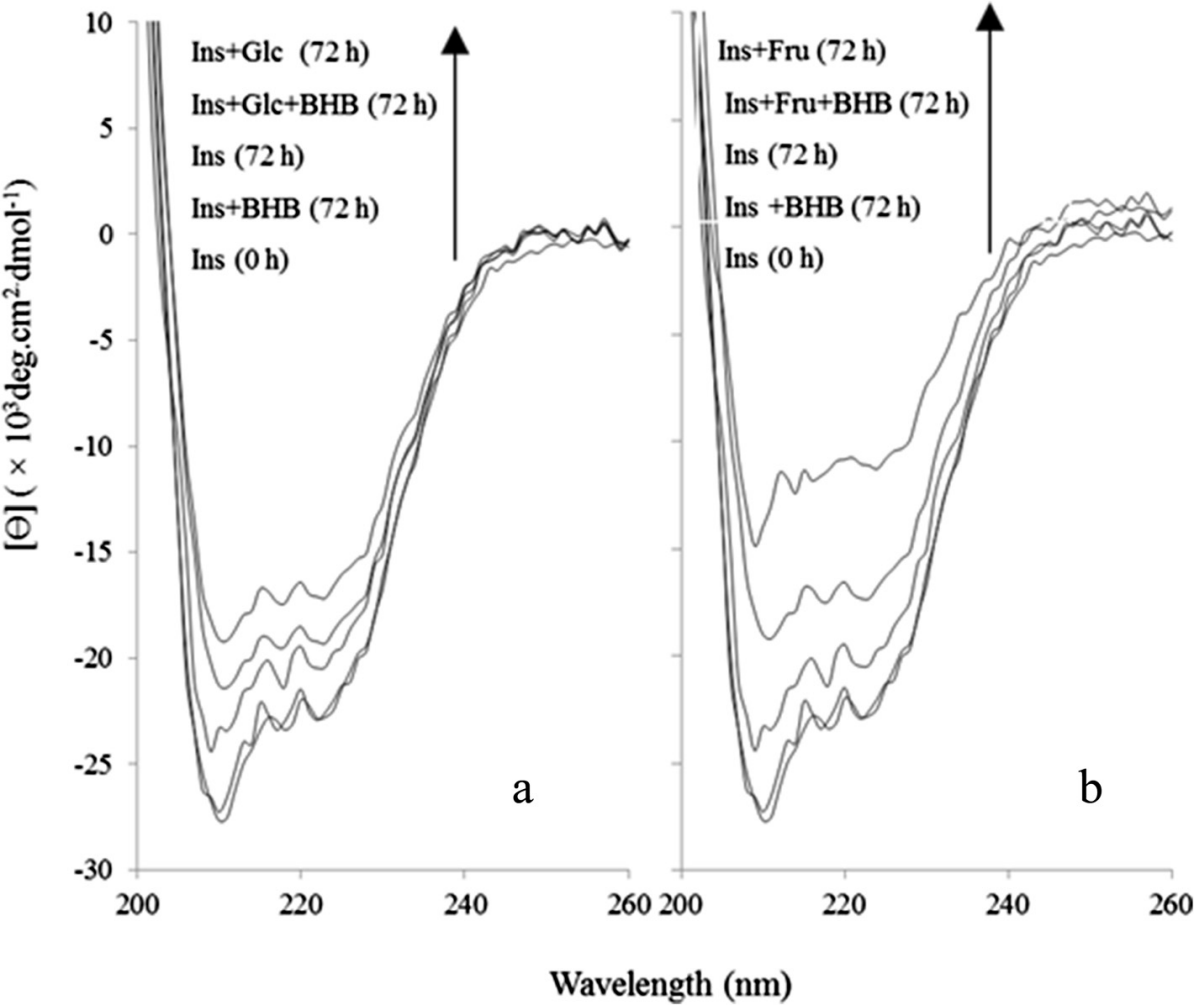
3-β hydroxybutyrate (BHB) inhibits the secondary structure change in glycated protein. **a** The secondary structure percentage of insulin and modified insulin (Ins + Glc, Ins + Glc + BHB) in 50 mM sodium phosphate buffer pH 7.4 containing 0.1 mM sodium azide incubated at 37 °C for 72 h. **b** CD spectra of insulin glycated with fructose in the presence or absence of BHB

Figure 2 represents glycation-induced insulin secondary structure transformation by Glc (2a) or Fru (2b), using circular dichroism (CD) for the products of 72 h incubation; inhibitory effect of BHB was also included. Glycation by Glc or Fru brings about 9.7 and 15.1 percent decrease in α-conformation, respectively. BHB not only inhibited all, 7.9 % decreasing in α-conformation due to glycation by Glc, but also caused even 0.9 % increasing of this conformation for insulin incubated alone for 72 h (BHB treatment similarly diminished increase in β-conformation from 3.82 % to 0.2 %). Under glycation by Fru, BHB diminished α-decrease or β-increase from 13.3 % to 6.7 % and 8.12 % to 2.8 %, respectively (Table 1). These results support a protective effect of BHB against glycation-induced sheet formation, proceeding less fibril formation, and indicates on a structural inertia to glycation induced conformational changes due to the BHB.

**Table 1:** The relative percentages of the secondary structures were estimated using the CDNN CD spectra deconvolution software. The results are expressed as mean ± S.D. from three independent experiments

Sample	α-Helix	β-Sheet	β-Sheet	β-Turn	Random-coil
Ins 0 h	39.2 ± 0.2	5.8 ± 0.05	7.7 ± 0.03	15.5 ± 0.0	31.4 ± 0.06
Ins 72 h	37.4 ± 0.1	6 ± 0.1	8 ± 0.03	15.5 ± 0.09	32.7 ± 0.3
Ins + Glc 72 h	29.5 ± 0.2	8.42 ± 0.1	9.4 ± 0.05	16 ± 0.03	36.4 ± 0.1
Ins + Fru 72 h	24.1 ± 0.05	10.5 ± 0.3	11.7 ± 0.5	16.7 ± 0.3	38.1 ± 0.9
Ins + BHB 72 h	36.3 ± 0.1	732 ± 0.2	8.1 ± 0.2	16.1 ± 0.1	32.0 ± 0.08
Ins + Glc + BHB 72 h	38.3 ± 0.1	5.9 ± 0.1	7.8 ± 0.03	15.5 ± 0.05	32.0 ± 0.08
Ins + Fru + BHB 72 h	30.7 ± 0.08	7.6 ± 0.8	9.2 ± 0.03	15.7 ± 0.03	36.5 ± 0.08

### Lipid peroxidation analysis

**Fig. 3:**
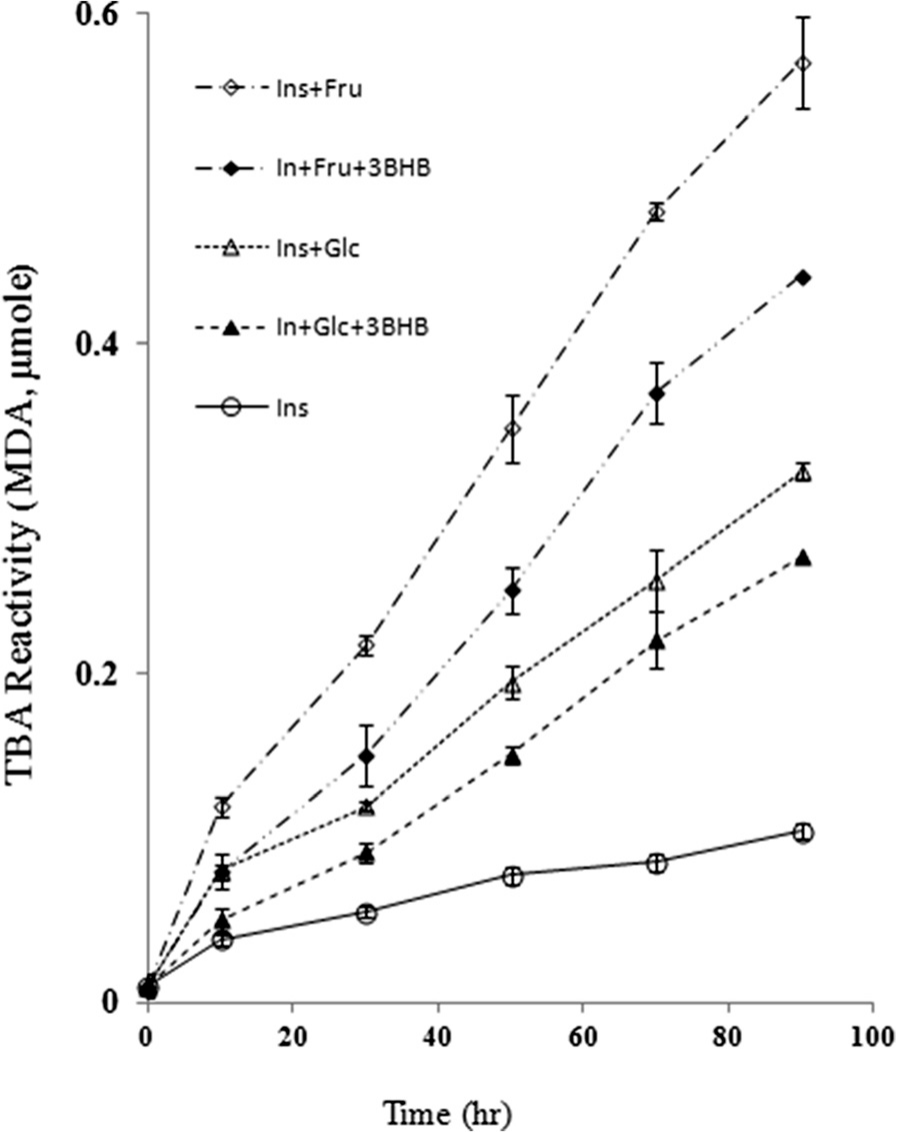
The liposomal lipid peroxidation derived by insulin glycation and reduced by BHB. It was measured using TBA assay based on MDA and TBARS monitoring. MDA formation was determined at 532 nm against the time of incubation of glycated insulin by Glc or Fru in the presence or absence of BHB. Results are expressed as mean ± S.D. from three independent experiments

In order to investigate the preventive effect of BHB on lipid peroxidation potential, TBARs assay was performed. Figure 3 shows the level of lipid peroxidation marker, malondialdehyde (MDA) at 532 nm as a function of time. The level of MDA as an end product of lipid peroxidation was markedly increased in the presence of insulin glycation by glucose or fructose that was effectively inhibited by BHB (Fig. 3), most probably or as a reason of preventive effect of BHB on insulin glycation or pertained antioxidative property.

### Microglial cell survival assay

**Fig. 4:**
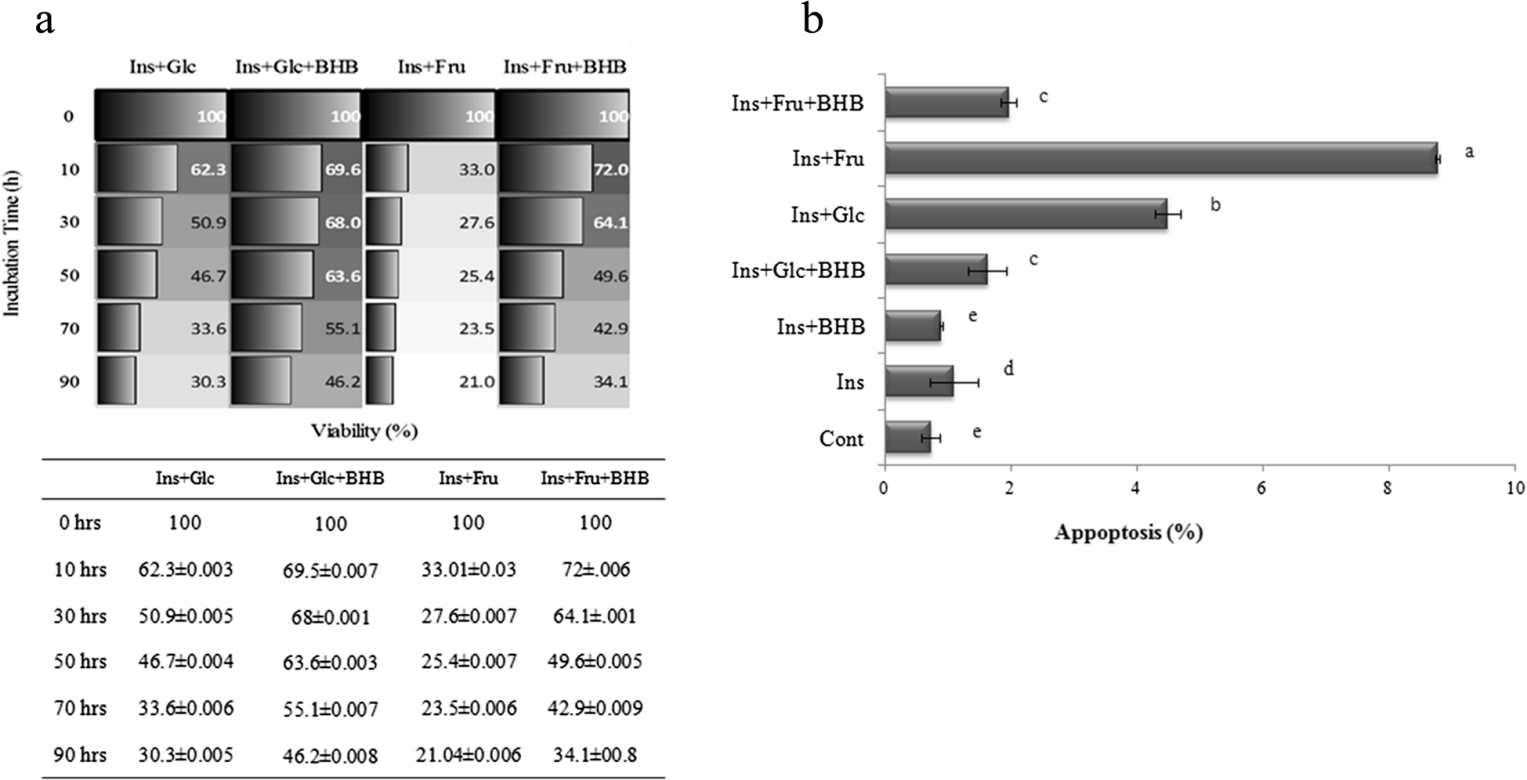
β-hydroxybutyrate (BHB) reduced cytotoxicity of glycated proteins on microglial cells. **a** Cell viability measured by MTT assay. Insulin alone or incubated with Glc or Fru in the presence or absence of BHB at 37 °C for 10, 30, 50, 70, and 90 h, were added to microglial cells for 24 h. Cell viability was measured using MTT assay and absorbance of the solutions was measured at 540 nm. Results are expressed as mean ± S.D. from five independent experiments, (**b**) Microglial cells were treated for 24 h with 72 h glycated insulin glycated in the presence or absence of BHB. In controls, insulin was incubated without Glc or Fru, in the presence or absence of BHB. The rate of apoptosis of treated microglia was detected by Annexin-V apoptosis Assay kit and analyzed by flow cytometry. Results are expressed as mean ± S.D. from three independent experiments. Treatments with different letters at the top of the bars are significantly different from each other according to analysis of variance (*P <* 0.01)

Random images were obtained and semi-confluent ramified microglial culture was observed to confirm the right phenotype of the isolated cells. The effect of insulin-AGEs as the products of insulin glycation by glucose or fructose on rat microglial viability was studied using MTT assay according to the conventional protocols with at least 5 repeats [23]. As shown in Fig. 4a, when culture medium was replaced with medium supplemented with products of insulin glycation in the absence of BHB for different period of time (0, 10, 30, 50, 70 and 90 h), the cell viability was dramatically affected by the presence of insulin–AGEs and as depicted, fructation derived insulin-AGEs were 1.4–1.8 folds more effective than the glycation products of Glc on decreasing cell viability in the absence of BHB (Fig. 4a). However, in the presence of BHB the cell viability was improved at more than 1.5 folds. Besides, the indices of rat microglial apoptosis were determined using Annexin V/PI staining [29]. The flow cytometry (FACS) analysis was carried out on microglial cells that were treated with glycated insulin for 72 h in the presence or absence of BHB against corresponding control (Fig. 4b). The percentage of cell apoptosis were significantly increased when microglial culture was treated with insulin glycation products, especially by fructose BHB decreased apoptotic cells about 5.3 and 8.2 folds corresponding to the cells treated with glycation products of glucose or fructose, respectively.

## Discussion

Nowadays, protein aggregation diseases such as Alzheimer’s, Parkinson’s, cataract, mad cow diseases and physiological aging have attained particular attention. Moreover, protein aggregation turns out to play a significant role in cancer i.e. p53 aggregation (as an important tumor suppressor protein) leads to uncontrolled cell growth [30]. One of the causative conditions in protein aggregation known to be protein glycation [31] which not only makes friend proteins disabilities, but also makes them toxic and foe. In protein glycation after a nonenzymatic reaction between reducing sugars and protein, a series of glycation products are consecutively generated, including soluble prefibriles and non-soluble fibrils that are collectively called advance glycated end products or AGEs. Considerable evidences indicate that under hyperglycemia, protein glycation and AGE generation are important determinants of complications often observed in type 1 and type 2 diabetes, including nephropathy, retinopathy, neuropathy and cardiovascular disease (CVD) [32]. Although, almost all proteins could be targets for glycation, but more specifically, concurrency of hyperglycemia and hyperinsulinemia in type 2 diabetes makes insulin prone to be glycated to generate ROS and produce insulin-AGEs. Insulin is glycated even in the pancreas which has been considered in the pancreas of various animal models of type 2 diabetes [33, 34]. Prevention of protein glycation and its consecutive symptoms are of great importance. We are reporting the inhibitory effect of BHB on insulin-AGE formation and insulin-AGE toxicity in microglial cells. When insulin was incubated in the presence of glucose or fructose, insulin was glycated and AGE-dependent fluorescence was increased by the time, followed by a protein conformational change monitored by decreasing Tyr or increasing ANS fluorescence intensities; while BHB inhibited all three mentioned cases. Since most AGEs (such as N-carboxymethyl lysine; CML and N-carboxyethyl lysine; CEL, and cross links such as pentosidine, methylglyoxal lysine dimmer; MOLD, and threosidine) have a characteristic fluorescence, with an excitation maximum at 360, and emission around 460 nm, detection through fluorescence spectroscopy is a widely available method. Insulin-AGEs formation and inhibitory effect of BHB were collectively monitored using AGEs fluorescence (ex360 nm, em460 nm) as a result of insulin incubation in the presence of Glc or Fru in Fig. 1a. Insulin has three glycation prone positions, the N-terminals of both chains (Gly1 and Phe1) and residue Lys29 of B-chain. As a result, three forms of insulin glycation products have been reported (mono, di, and tri-glycated forms) [35]. Most probably, the effect of BHB on preventing insulin glycation is due to BHB binding to glycation prone residues in protein to diminish the glycation susceptibility. In addition, proteins present their intrinsic fluorescence because of their main flourophore residues; tryptophan (Trp, W), tyrosine (Tyr, Y) and phenylalanine (Phe, F), but only Trp and Tyr are used experimentally because their quantum yields are high enough to give a good fluorescence signal [36]. In insulin as a special case, Tyr dominates the fluorescence excitation at 280 nm in the absence of Trp. It has been found that insulin denaturation results in (or brought about) a decrease in Tyr fluorescence, suggesting that in insulin, Tyr residues were translocated from hydrophobic pockets to the aqueous environment and were effectively quenched [37]. As a result of glycation process over the period of 96 h, further fluorescence quenching was observed when insulin was incubated in the presence of Glc and far more in the presence of Fru. Such conformational changes were also monitored using ANS fluorescence (Fig. 1c). The presence of BHB interestingly and successfully protected insulin against glycation-involved conformational changes. Insulin glycation ended in protein fibrillation that exhibited increased β-sheets (Fig. 1d) and decreased α-conformation (and increased β-conformation) (Fig. 2) relative to the non-glycated and non-fibrillar form reporting by CD analysis and ThT binding fluorescence, respectively. The CD is a powerful tool for investigating glycation dependent α- to β-conformational changes in proteins [38]. However, ThT can bind to β-sheets due to its geometric fitness [39], stochiometrically emits after excitation at 440 nm. As expected, Fru changed secondary structure and developed β-sheet more effectively than the Glc; however, the preventive effect of BHB was observed in both cases. Glycation has reported to cause a decrease in α-helix content in various proteins, e.g. human serum albumin (HSA) [40], bovine serum albumin (BSA) [41], and hemoglobin (Hb) [42]. More recently, we observed that BHB can preserve the secondary structure of HSA against Glc using CD [43]. Interestingly, BHB not only prevents glycation—derived sheet development, but also presents a stabilizing effect on insulin in the absence of Glc or Fru (Fig. 1d). Our results show that insulin-AGEs induce liposomal lipid peroxidation in a time dependent manner, nonetheless, BHB can effectively reduce these effects (Fig. 3). The relationship between the level of glycated hemoglobin and lipid peroxidation in erythrocytes of both diabetic and healthy subjects have been reported [44] and higher lipid peroxidation in seminal plasma of diabetic than non-diabetic subjects has been reported [45]. We assume that insulin glycation not only can explain insulin resistance, but also can play a role in cell death through lipid peroxidation more importantly glial cells. Also, we are reporting the anti-lipid peroxidation effect of BHB that can explain its protection on microglial apoptosis. To continue, the effects of products of insulin glycation by glucose or fructose on rat microglial survival as well as the protective effect of BHB on microglial cells were estimated using MTT assay and flow cytometry using Annexin V/PI staining.

These observations confirmed the cytotoxic effects of insulin glycation products, especially by fructose, on microglial cells (Fig. 4). Since microglia are implicated in cascades causing neuronal loss and cognitive decline in Alzheimer’s disease (AD), insulin–AGEs formation, especially in type 2 diabetes, are most probably involved in AD that is proposed as type 3 diabetes. However, BHB diminished the extent of toxicity evolved by insulin glycation on microglial cells or probably inhibited microglial cell apoptosis. Shan Chenga et al. has reported preventive effect of BHB on apoptotic and necrotic cell death by serving as a metabolic fuel for cells [46]. Because of the fact that BHB can cross the blood brain barrier (BBB), arriving at neurons and glial cells [47], the inhibitory effect of BHB on insulin-glycation, insulin-AGEs formation, and insulin-AGEs derived liposomal lipid peroxidation are collectively offer a possible explanation on protective effect of BHB on microglial apoptosis under diabetic condition.

## Conclusion

Insulin glycation by Glc and more effectively by Fru results in insulin-AGEs formation and lipid peroxidation that may involve in insulin resistance. Moreover, the toxic effect of insulin-AGEs on cultured microlia are reported in which, the glycation product of insulin by Fru presented more toxic effect than glycation product of insulin by Glc. Microglial apoptosis may involved in neurodegenerative diseases such as AD. The observed protective effects of BHB in insulin glycation and insulin-AGEs induced microglial apoptosis, disclose the new insights of BHB action against type 3 diabetes or AD.
